# Interstitial Molar Ectopic Pregnancy Despite Euploid Embryo Transfer: A Case Report

**DOI:** 10.1097/og9.0000000000000215

**Published:** 2026-11-10

**Authors:** Karishma Patel, Denny Sakkas, Yvette Groszmann, Lauren Murphy, Alan Penzias, Joseph Dottino

**Affiliations:** Department of Obstetrics and Gynecology, Beth Israel Deaconess Medical Center, Boston, Massachusetts; Boston IVF-IVIRMA Global Research Alliance, Waltham, Massachusetts; and Department of Obstetrics, Gynecology, and Reproductive Medicine, Harvard Medical School, Boston, Massachusetts.

## Abstract

**BACKGROUND:**

Even with modern in vitro fertilization (IVF) techniques, unexpected pregnancy outcomes can occur. Molar pregnancy after IVF and embryo transfer can be the result of true abnormal fertilization in the embryology lab. Preimplantation genetic testing for aneuploidies (PGT-A) has advanced and is widely used, but unless additional testing of single-nucleotide polymorphisms is performed, the rare case of partial molar pregnancy (defined as triploidy of paternal origin) after IVF may be missed. Unexpected pregnancy outcomes may also be a result of spontaneous fertilization during assisted reproductive technology (ART) treatment.

**CASE:**

The patient is a 36-year-old G1P100 who initially underwent IVF and intracytoplasmic sperm injection with PGT-A and preimplantation genetic testing for monogenic disorders for galactosemia carrier status in both partners. After obtaining euploid and galactosemia unaffected embryos, she underwent a frozen embryo transfer of a female, euploid embryo. The result was a right interstitial molar ectopic pregnancy that was managed with laparoscopic right cornual resection.

**CONCLUSION:**

Assisted reproductive technology with PGT-A attempts to eliminate the risk of abnormal embryo transfer. Technical factors, such as variations in testing processes among PGT-A laboratories, and human factors, such as unprotected intercourse during ART, contribute to unexpected pregnancy outcomes.

## Abstract 2

Assisted reproductive technology does not eliminate occurrence of molar pregnancy, which may be attributed to limitations of preimplantation testing and possible spontaneous fertilization with natural cycle assisted reproductive technology protocols.

**Teaching Points**

1. The risk of molar pregnancy is not eliminated with the use of assisted reproductive technology.
2. Preimplantation genetic testing for aneuploidies (PGT-A) only screens for large gains or losses of chromosome material, and does not always screen for all deletions, duplications, molar pregnancy, or other genetic conditions. Additional testing of single nucleotide polymorphisms can help diagnose molar pregnancy, but this is not routinely included in PGT-A testing algorithms, depending on the laboratory.
3. Patients should be counseled to abstain from unprotected intercourse during fertility treatment to mitigate the risk of unplanned outcomes associated with spontaneous fertilization, especially in the setting of hyperstimulated ovaries or in the setting of natural cycle embryo transfer.

Although many patients achieve successful outcomes with assisted reproductive technology (ART), there are cases that remind us that ART has limitations and comes with inherent risks. In vitro fertilization (IVF) with intracytoplasmic sperm injection (ICSI) should, theoretically, be protective against molar pregnancy given that the pathogenesis commonly involves abnormal fertilization with two sperm (dispermic fertilization).^1^ Cases still exist after ICSI, however, with one study reporting that the incidence of molar pregnancy after ICSI was 1 of 4,302 in their cohort.^2–4^ At the same time, preimplantation genetic testing for aneuploidies (PGT-A) can screen for large gains and losses in chromosomes, but, alone, it cannot screen for all genetic conditions, including molar pregnancy.

In the context of ART, spontaneous fertilization is still possible during IVF, with rare case reports demonstrating as such.^5,6^ The rise of natural cycle frozen embryo transfer cycles, especially, introduces the risk of spontaneous fertilization given ovulation occurs. Indeed, molar pregnancy can also occur in the setting of IVF or frozen embryo transfer if unprotected intercourse is not avoided.

The following case is of an interstitial molar ectopic pregnancy after a frozen embryo transfer of a euploid embryo tested for PGT-A. While the exact mechanism is unknown, it allows an opportunity to explore the limitations of controlled ART procedures such as ICSI, PGT-A, and frozen embryo transfer. We confirm that written consent has been obtained by the patient described in the case report.

## CASE

A 36-year-old G1P1001 initially presented to undergo IVF with preimplantation genetic testing for monogenic disorders, given both parents were carriers for galactosemia. The couple elected for the embryos to also undergo PGT-A. The patient and partner were healthy, and fertility evaluation revealed no abnormalities.

The couple underwent IVF with ICSI, which resulted in three euploid embryos that were unaffected by galactosemia. Frozen embryo transfer cycles were initiated and were notably complicated by thin endometrial lining, causing cycle cancellations. The patient's fourth attempt was a natural cycle frozen embryo transfer, which yielded a lining of 5.3 mm, and a transfer of a euploid, female embryo unaffected by galactosemia. This resulted in a positive β-hCG (initial β-hCG was 236 mIU/mL 10 days after transfer and was 1960 mIU/mL when repeated 4 days later ). A clinic ultrasound at 7 weeks of gestation showed a vascular endometrium with a small complex collection that could not be distinguished as within the endometrium (Fig. 1). On the day of this ultrasound, the β-hCG was 85,965 mIU/mL. The next day, a follow-up ultrasound at a specialized obstetrics and gynecology ultrasound clinic showed a 31×27×29 mm hyperechoic mass in the right interstitial portion of the uterus, with moderate-to-marked color flow with appearance suspicious for molar pregnancy (Fig. 2). The patient then underwent an uncomplicated diagnostic hysteroscopy, diagnostic laparoscopy, excision of interstitial ectopic pregnancy from the right uterine cornua, and right salpingectomy. Intraoperative findings were notable for an enlarged bulge of right uterine cornua; upon incision into the cornua, ectopic tissue appeared to be placental-like, consistent with suspected molar pregnancy (Fig. 3). Final pathology revealed chorionic villi with morphological features compatible with partial mole.

**Fig. 1.**
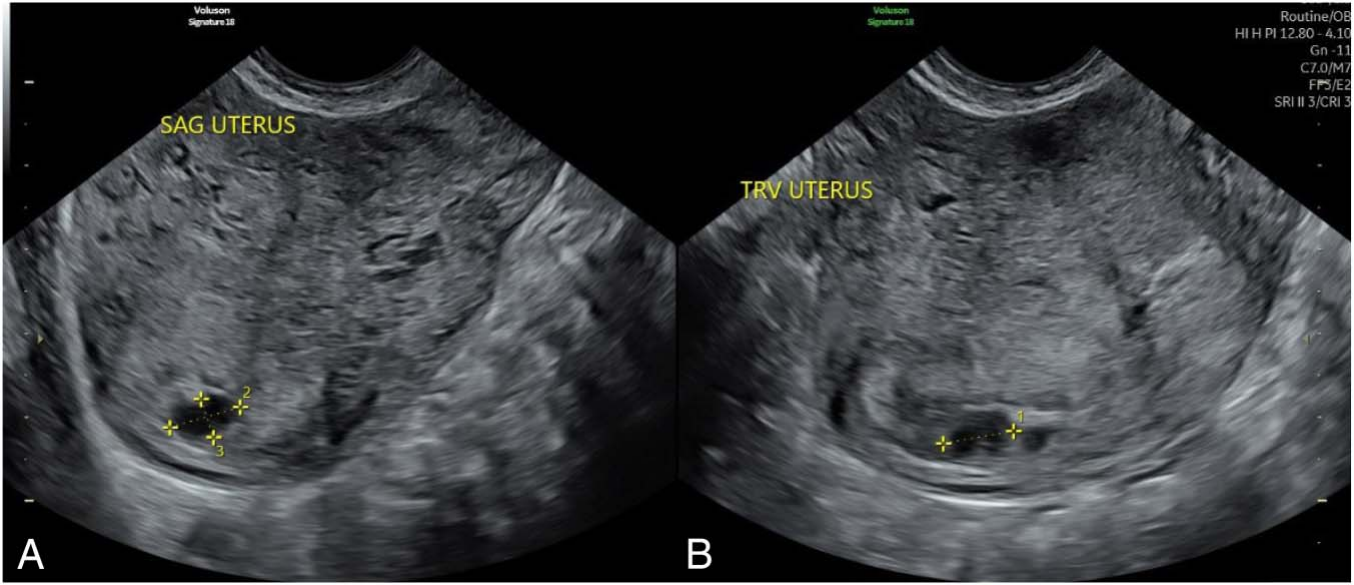
Ultrasonogram images performed at fertility clinic at 7 weeks of gestation. Sagittal (**A**) and transverse (**B**) views of the uterus. Calipers are measuring the complex collection that was not able to be identifiable as part of the endometrium.

*Patel. Molar ectopic after euploid embryo transfer. O&G Open 2026*.

**Fig. 2.**
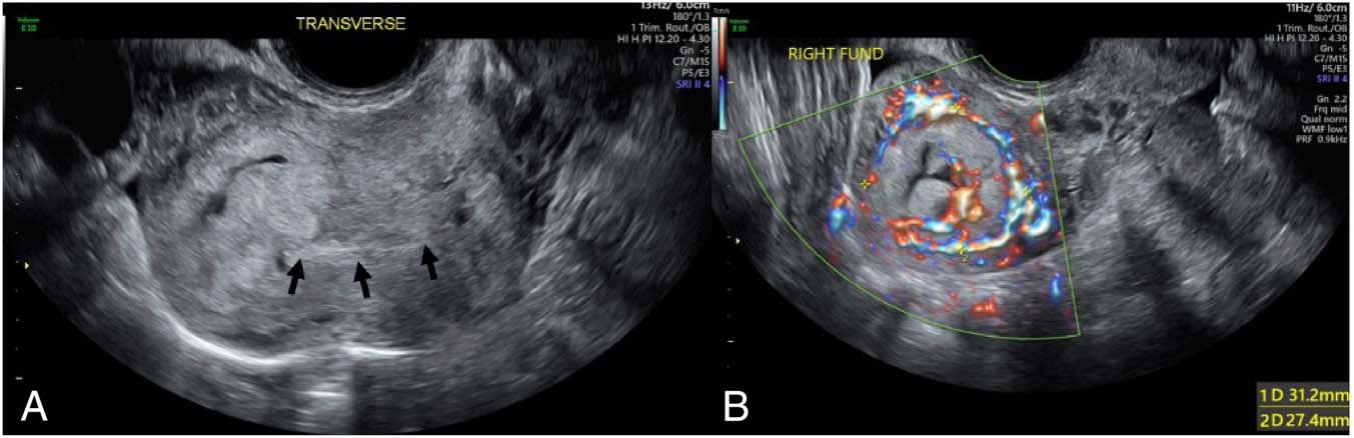
Ultrasonogram images performed at a specialized ultrasound clinic at 7 weeks of gestation. **A**. Transverse view of the uterine fundus and shows a hyperechoic mass in the right interstitial portion of the uterus. The separate, thin endometrium is marked by *black arrows*. **B**. Interstitial mass with color Doppler assessment in the sagittal plane.

*Patel. Molar ectopic after euploid embryo transfer. O&G Open 2026*.

**Fig. 3.**
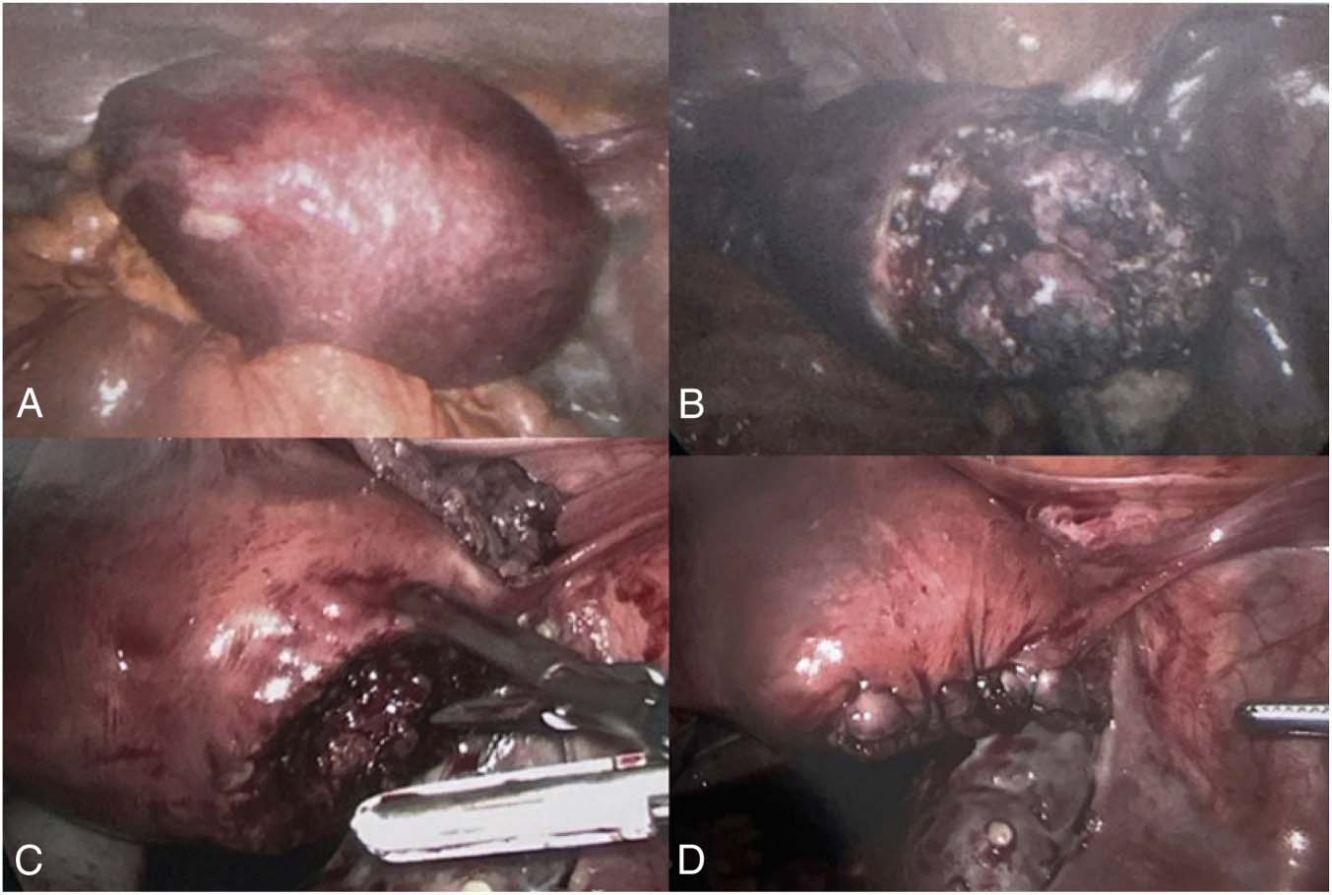
Intraoperative pictures. Initial appearance of the uterus upon pelvic survey (**A**), appearance of ectopic tissue after incision into the cornua (**B**), right cornual defect after removal of ectopic tissue (**C**), and closed cornual defect (**D**).

*Patel. Molar ectopic after euploid embryo transfer. O&G Open 2026*.

## DISCUSSION

Molar pregnancy is estimated to affect approximately 1 in 1,000 pregnancies.^7^ Partial molar pregnancy is characterized by triploidy of paternal origin. In 95% of cases, this occurs when an egg is fertilized by two sperm, resulting in 69 XXX or 69 XXY genotype.^8^ In the remaining cases, partial mole can occur when an egg is fertilized by one sperm that has a duplicated genome.^8^ Specific to this case, two possible mechanisms for partial molar pregnancy will be discussed. One possibility is that an embryo with true partial molar genotype was created in the IVF laboratory and the PGT-A screen, given its limitations as a test, could not identify this pathology. Another possibility is that the euploid embryo actually did not implant, and rather a spontaneous molar interstitial pregnancy occurred during the natural frozen embryo transfer cycle.

In the context of ART, ICSI precludes dispermic fertilization. Partial molar genotype of the embryo is still possible if the single sperm selected is diploid, meaning the sperm contains two copies of paternal DNA due to meiotic error.^1^ When a diploid sperm is injected into a haploid oocyte, the resulting genotype is triploid with two paternal sets and one maternal, resulting in partial molar pathology. Diploid sperm are rare and only present in 0.2% of healthy men, and 1–2% of men with fertility problems,^1,2^ thus there are no routine screening methods to detect diploid sperm before ICSI.

If partial molar pregnancy does occur after IVF with ICSI, PGT-A may only provide an limited risk assessment, depending on the testing utilized. Most PGT-A laboratories utilize next-generation sequencing on a 5–10 cell biopsy of the embryo's trophectoderm (the outer layer that becomes the placenta).^9^ The extracted DNA is first amplified and then fragmented so that unique sequencing adapters can be ligated to the DNA; these unique adapters correlate to specific sequences found on each distinct chromosome. Next-generation sequencing platforms (which vary across PGT-A laboratory) are used to read the DNA, which then undergoes bioinformatic analysis. Bioinformatic analysis creates an amplification map, and aneuploidy is identified if amplification of a certain chromosome sequence is significantly different from the remainder of the output (refer to Capalbo et al^10^ for further methodological information). Thus, PGT-A only screens for large gains or losses of chromosome material, and does not screen for all deletions, duplications, uniparental disomy, or other genetic conditions. For example, complete duplication or deletion of a full chromosome set will not be identified by next-generation sequencing, alone, since the bioinformatics map will not show any amplification abnormality relative to the whole output. In this patient's case, the PGT-A result indicated female (no amplification of Y chromosome sequences) and euploid (no abnormal variations in amplification relative to the whole output) (Fig. 4).

**Fig. 4.**
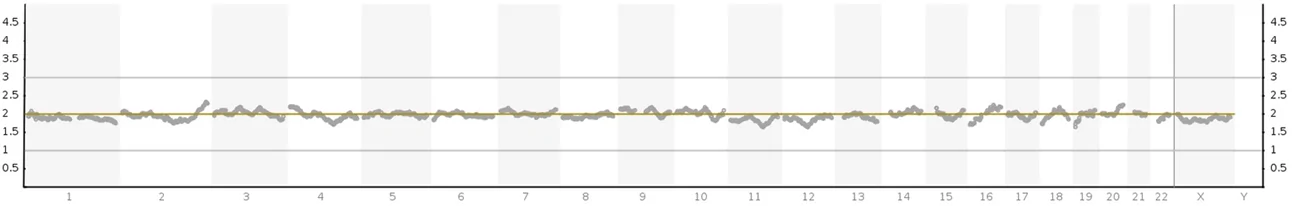
Preimplantation genetic testing for aneuploidies output graph for the embryo transferred. Amplification occurred at the same level for all chromosomes indicating no duplication or deletion leading to euploid result of the embryo. Determination of ploidy status and paternal contribution requires additional testing.

*Patel. Molar ectopic after euploid embryo transfer. O&G Open 2026*.

An important limitation of PGT-A testing, as previously described, is the inability to determine parental contribution of genetic material. Determining this involves additional preimplantation testing of single nucleotide polymorphisms (SNPs), which may not be universally included in a PGT-A laboratory's testing platform. Single nucleotide polymorphisms represent the most common form of genetic variation among individuals, serving as their unique fingerprint. Without SNP testing, there is an inability to potentially recognize a SNP allelic pattern consistent with partial mole (two paternal contributions and one maternal contribution). Cases like this reinforce that PGT-A is ultimately only a screening tool with inherent potential for false negative and false positive results; thus, it is still recommended to complete prenatal aneuploidy screening or diagnostic testing in early pregnancy, even after a transfer of a euploid embryo as determined by PGT-A testing.^11^

Regarding the other possible mechanism, the risk of spontaneous fertilization during a frozen embryo transfer cycle exists given that some protocols follow the natural menstrual cycle. In natural cycle frozen embryo transfers, spontaneous or medically triggered ovulation occurs, and the embryo is transferred based on this timing. Favorable aspects of natural cycle frozen embryo transfers include minimal medication and the presence of a corpus luteum, which has been increasingly recognized as beneficial for pregnancy and obstetric outcomes.^12^ Patients are counseled to use barrier contraception or abstain from intercourse during these cycles, given that the oocyte released is potentially fertilizable. In this case, the patient presented for preimplantation genetic testing for monogenic disorders and was not infertile, putting her at higher risk, and the timing of any potential unprotected intercourse is unknown. Unprotected intercourse during ART can result in spontaneous healthy pregnancy, multiple pregnancy, or even heterotopic pregnancy if the embryo were to also implant.

Another complex aspect was the interstitial implantation. In a large study of 10,143 embryo transfer (ET) cycles, the incidence of interstitial ectopic was 0.4% per ET cycle.^13^ Of all ectopic pregnancies diagnosed after ET, 35.5% were interstitial versus only 2–4% of ectopic pregnancies in the general population that were interstitial.^13,14^ Embryos have an inherent ability to migrate; studies show that embryos implant at the exact transfer location 40–50% of the time.^15^ Other factors contributing to ectopic implantation include uterine contraction, suboptimal endometrial receptivity, and thin endometrial lining.^16,17^ Given this rare outcome, we did not identify literature suggesting an increased likelihood of interstitial implantation of molar pregnancies following IVF or spontaneous pregnancy.

While early interstitial pregnancy may be managed medically or surgically, molar pregnancy requires evacuation given the hemorrhage risk, failure rates without surgical intervention, and risks of gestational trophoblastic neoplasia; thus, definitive surgical evacuation was performed as previously described. Postoperative management was informed by Society of Gynecologic Oncology guidelines, which included following β-hCG levels weekly until normalization and performing one additional hCG a month after normalization.^18^ Subsequent pregnancy attempts are safe once β-hCGs resolve, with a recurrent molar pregnancy risk of 1–2%.^19,20^ In this case, future frozen embryo transfers would be delayed for 3–6 months to allow uterine healing.

Overall, partial molar pregnancy can still occur despite normal PGT-A results, as PGT-A, alone, cannot screen for triploidy of paternal origin. Partial molar pregnancy can also happen if spontaneous fertilization occurs around the time of frozen embryo transfer. This is demonstrated by the subject case in which a partial molar ectopic pregnancy resulted after IVF with ICSI with normal PGT-A results.
